# Mungo bean sprout microbiome and changes associated with culture based enrichment protocols used in detection of Gram-negative foodborne pathogens

**DOI:** 10.1186/s40168-016-0193-y

**Published:** 2016-09-06

**Authors:** Heike Margot, Roger Stephan, Taurai Tasara

**Affiliations:** Institute for Food Safety and Hygiene, Vetsuisse Faculty University of Zurich, Winterthurerstrasse 272, 8057 Zurich, Switzerland

**Keywords:** Microbiome, Sprouts, Enterobacteriaceae enrichment broth, Buffered peptone water

## Abstract

**Background:**

Fresh sprouted seeds have been associated with a number of large outbreaks caused by *Salmonella* and Shiga toxin-producing *E. coli*. However, the high number of commensal bacteria found on sprouted seeds hampers the detection of these pathogens. Knowledge about the composition of the sprout microbiome is limited. In this study, the microbiome of mungo bean sprouts and the impact of buffered peptone water (BPW) and Enterobacteriaceae enrichment broth (EE-broth)-based enrichment protocols on this microbiome were investigated.

**Results:**

Assessments based on aerobic mesophilic colony counts showed similar increases in mungo bean sprout background flora levels independent of the enrichment protocol used. 16S rRNA sequencing revealed a mungo bean sprout microbiome dominated by *Proteobacteria* and *Bacteroidetes*. EE-broth enrichment of such samples preserved and increased *Proteobacteria* dominance while reducing *Bacteroidetes* and *Firmicutes* relative abundances. BPW enrichment, however, increased *Firmicutes* relative abundance while decreasing *Proteobacteria* and *Bacteroidetes* levels. Both enrichments also lead to various genus level changes within the *Protobacteria* and *Firmicutes* phyla.

**Conclusions:**

New insights into the microbiome associated with mungo bean sprout and how it is influenced through BPW and EE-broth-based enrichment strategies used for detecting Gram-negative pathogens were generated. BPW enrichment leads to *Firmicutes* and *Proteobacteria* dominance, whereas EE-broth enrichment preserves *Proteobacteria* dominance in the mungo bean sprout samples. By increasing the relative abundance of *Firmicutes*, BPW also increases the abundance of Gram-positive organisms including some that might inhibit recovery of Gram-negative pathogens. The use of EE-broth, although preserving and increasing the dominance of *Proteobacteria*, can also hamper the detection of lowly abundant Gram-negative target pathogens due to outgrowth of such organisms by the highly abundant non-target *Proteobacteria* genera comprising the mungo bean sprout associated background flora.

## Background

In the last decades, sprouted seeds have become a popular food item. They are available year round and are usually consumed raw. The production of sprouted seeds follows a complex path from the farm to the consumer including the production and shipping of the seeds followed by sprouting and distribution of the finished product. Since conditions during sprouting are warm (20 to 25 °C) and humid, the sprouting process is a potent bacterial amplification step occurring shortly before the packaging and consumption [1]. Although normal flora of sprouted seeds is usually not a threat to human health, contamination with foodborne pathogens can occur at many points during production subsequently leading to human infections [2, 3]. Aerobic bacterial counts as high as 10^7^ to 10^9^ cfu/g and the presence of foodborne pathogens such as *Salmonella*, Shigatoxin-producing *Escherichia coli* (STEC), and *Listeria monocytogenes* have been reported in alfalfa, fenguk, and mungo bean sprouts samples collected at retail level in different countries [2–7]. Moreover, sprouted seeds contaminated with enteric pathogens have been associated with many large illness outbreaks, most of which have been linked to *Salmonella* and STEC [3, 8, 9]. One of the biggest outbreak that occurred in 2011 in Germany during which more than 50 people died was due to fenugreek sprouts most likely contaminated with Shigatoxin-producing *E. coli* O104:H4 [3]. An international outbreak of *S.* Newport infections occurred during 2011 in Germany and Netherlands following the consumption of contaminated mungo bean sprouts [8]. An outbreak due to the consumption of *S.* Enteritidis-contaminated mungo bean sprouts involved several states and caused 111 cases of illness during 2014 in the USA [9].

The high numbers and complexity of the non-pathogenic bacteria constituting the normal flora of sprouts makes this matrix a great challenge for microbiological detection methods. For example, despite substantial efforts during the German outbreak mentioned above the offending Shigatoxin-producing *E. coli* O104:H4 pathogen could not be detected in any of the samples from the fenugreek seeds and sprouts that were tested [3]. Currently, the standard method for STEC detection from sprouted seeds involves a non-selective enrichment step in buffered peptone water (BPW) before the detection of the *stx* genes by PCR [10]. The enrichment is supposed to increase the number of the target bacteria and thereby increasing the probability of detection. However, due to different factors such as competition with co-enriching sprout microflora, as well as differences in growth rates and presence of growth inhibitors, this enrichment does result in a biased sample [11]. In the case of sprouted seeds deliberately contaminated with STEC, we recently showed that growth of the target organisms terminates prematurely and PCR cannot reliably detect its presence even at high contamination levels [12]. Furthermore, attempts to increase the selectivity of the enrichment using different media or selective supplements did not have a significant impact on reducing the levels of co-enriching sprout background flora during enrichment [12].

In other studies, the impact of enrichment procedures on the phyllosphere microflora associated with tomatoes and cilantro has been investigated [13, 14]. However, for sprouted seeds, the identity of bacterial taxa co-enriched during enrichment procedures and the microbial dynamics influencing the detection of target pathogens are still poorly understood. The current study used 16S rRNA sequencing to examine the baseline microbiome composition in mungo bean sprouts and its changes during enrichment protocols used for the detection of STEC and other gram negative pathogens using BPW and EE-broth media cultured at 37 and 42 °C.

## Methods

### Sample preparation, bacterial enumeration, and community DNA isolation

Fresh mungo bean sprouts originating from the same batch obtained from a Swiss supermarket were used for the profiling of the phyllosphere bacteria; 10 g portions of sprouts diluted 1/10 in 0.9 % saline and homogenized in a Stomacher served as the non-enriched (*t* = 0) control samples. In addition, 10 g portions from the same batch diluted 1/10 in either Enterobacteriaceae enrichment broth (EE-broth, Oxoid CM0317) or buffered peptone water (BPW, Oxoid CM1049) were similarly homogenized, incubated at 37 and 42 °C and sampled after 4, 8, 16, and 24 h of incubation. The experiments were performed independently on three occasions. To enumerate the background flora, tenfold serial dilutions of 0.1 ml aliquots from each sample were prepared in 0.9 % saline and plated on tryptone soy agar (TSA, BD, Allschwil, Switzerland) plates and incubated for 24 h at 37 °C. To isolate microbial community DNA, 2 ml aliquots of the non-enriched and enriched sprouts suspension were centrifuged (10 min at 3200 *g*) to pellet sprout particles and debris. DNA was isolated from the cleared suspensions using the NucleoSpin® Soil kit (Macherey Nagel, Düren, Germany) according to the manufacturer’s instructions.

### Microbial profiling with Illumina HiSeq2500

Bacterial community composition of the samples was assessed by sequencing amplicons of the 16S rRNA gene. Sequencing was performed at GATC (Konstanz, Germany) based on their 300-bp paired-end protocol (https://www.gatc-biotech.com/de/produkte/inview.applikationen/inview-microbiome.html). Briefly, 471-bp fragments of the variable regions V1–V3 of the 16R rRNA gene in the samples were amplified using the primer pair 27F (AGAGTTTGATCCTGGCTCAG) and 534R (ATTACCGCGGCTGCTGG) and subjected to Illumina sequencing. Resulting 16S rRNA gene amplicons were quality filtered and merged based on overlapping bases using FLASh with maximum density of 0.25 [15]. The aligned merged filtered sequences were clustered into operational taxonomic units (OTUs) defined by 97 % similarity. The taxonomic status of the generated OTU clusters was assigned by BLAST against non-redundant 16S rRNA reference sequences obtained from the Ribosomal Database Project (RDP Release 11 updated September 2014) [16, 17]. The Shannon and Simpson diversities of the samples were calculated using the R package Vegan (http://cc.oulu.fi/~jarioksa/softhelp/vegan/html/diversity.html). Venn diagrams were constructed using Venny (http://bioinfogp.cnb.csic.es/tools/venny/index.html). The ß diversity between the non-enriched and enriched mungo bean sprout microbiomes was estimated using UniFrac and Bray-Curtis dissimilarity analysis implemented in CLC genomics Workbench (QIAGEN, Prismet, Denmark). Metastats, which is based on a nonparametric *t* test, Fisher’s exact test, and the false-discovery rate was used to determine OTUs that were statistically significantly different when groups were compared (*P* ≤ 0.05) [18].

## Results

### Growth dynamics of the sprout background flora

**Fig. 1:**
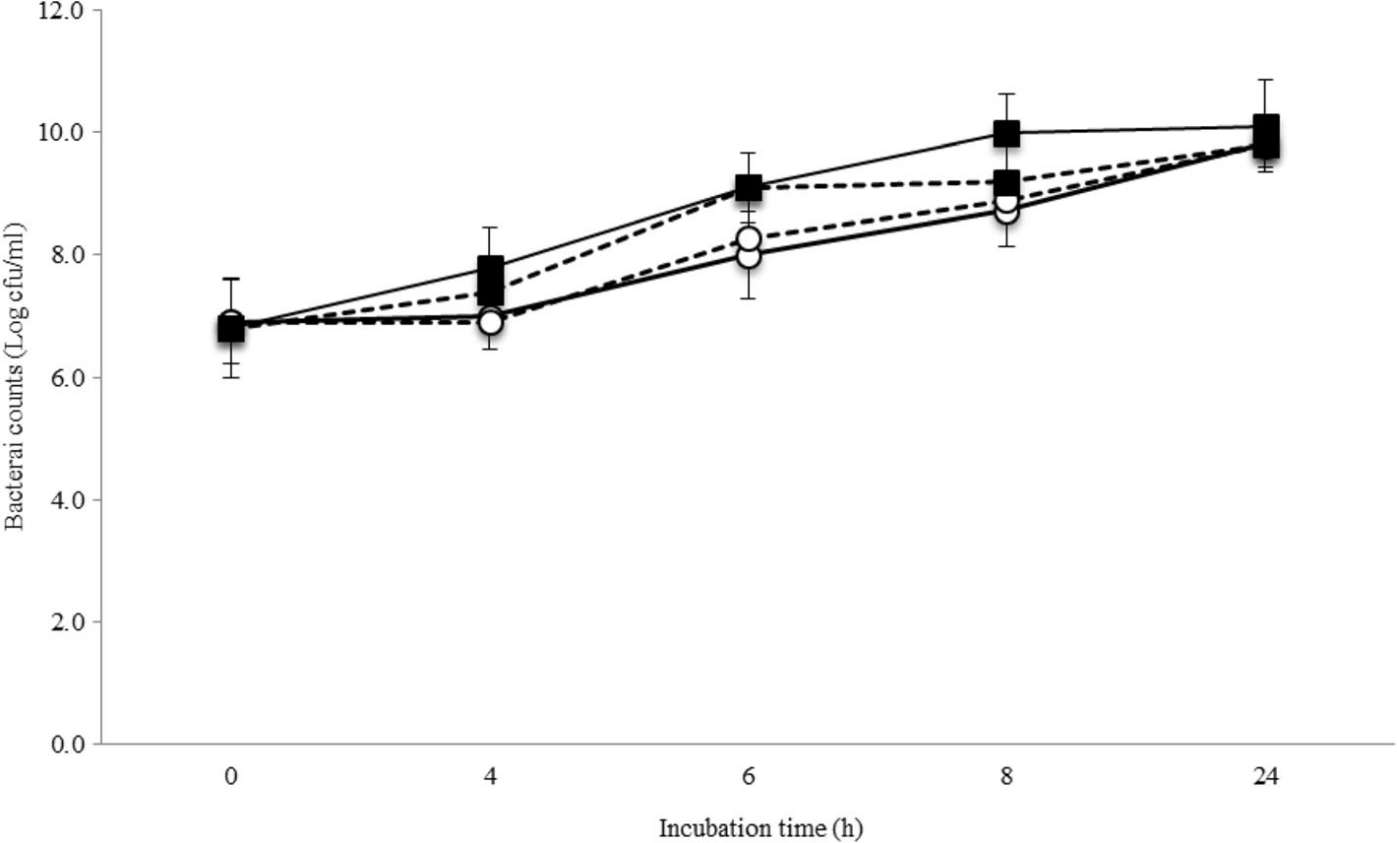
Enumeration of microorganisms on fresh mungo bean sprouts during incubation in BPW (*black square*) and EE-broth (*circle*) at 37 °C (*black line*) and 42 °C (*dashed line*). Total APC counts were determined. Means from two independent experiments are plotted, and standard deviations are indicated by *error bars*

The level of background flora on mungo bean sprout samples and its change over time during cultivation in BPW and EE-broth incubated at 37 and 42 °C were assessed based on total aerobic mesophilic counts (Fig. 1). Aerobic plate counts (APCs) determined showed that prior to culturing the mungo bean sprout samples harboured around 6.9 ± 0.81 log cfu/ml of mesophilic organisms that were dominated by *Klebsiella*, *Citrobacter*, and *Pantoea* species. Interestingly, the final level of microorganisms reached after a 24-h cultural enrichment were independent of either the enrichment media (BPW or EE-broth) or the incubation temperature (37 or 42 °C) used. In both enrichment media, the levels of mungo bean sprout background flora increased to reach similar levels in samples incubated at 37 °C (10.0 ± 0.75 log cfu/ml) and 42 °C (9.8 ± 0.85 log cfu/ml). Although at both incubation temperatures, the rate of background flora population growth was slightly faster in BPW compared to EE-broth.

### Sequencing results

Samples representing three independent biological replicates of uncultured mungo bean sprout samples as well as those cultured 4, 8, 16, and 24 h in BPW and EE-broth were subjected to deep sequencing of the 16S rRNA gene amplicons in order to get a more detailed view of the composition and microbial population dynamics in the mungo bean sprout associated flora during such culture-based enrichment protocols. Fifty-one DNA samples isolated from uncultured (*t* = 0) mungo bean sprout samples and at different stages (4, 8, 16, and 24 h) during enrichment in BPW and EE-broth samples cultivated at 37 and 42 °C were sequenced (Table 1); 8,301,847 quality filtered 16S rRNA sequence reads with an average of 162,781 reads (range 80,004–227,564) per sample were generated. Taxonomic assignment of these sequences based on a 97 % similarity definition of an operational taxonomic unit (OTU) generated 2169 OTUs with an average of 43 OTUs per sample.

**Table 1:** Sequencing reads and OTU metrics of the 51 non-enriched and enriched mungo bean sprout samples

Sample	OTU assigned reads	Phylum	Order	OTUs class	Family	Genus	Total
AT0	167,711	3	9	5	11	17	45
BT0	165,276	3	11	5	14	28	61
CT0	194,010	4	10	6	11	22	53
A4BPW37	170,966	3	10	5	12	21	51
B4BPW37	175,945	4	12	6	15	31	68
C4BPW37	197,930	3	7	4	8	18	40
A8BPW37	160,355	3	9	5	11	24	52
B8BPW37	143,794	3	10	5	12	28	58
C8BPW37	163,528	3	8	4	9	19	43
A16 BPW37	184,187	3	9	5	11	32	60
B16BPW37	110,423	2	6	4	7	20	39
C16BPW37	128,033	2	7	4	8	17	38
A24BPW37	124,741	3	12	7	16	32	70
B24BPW37	96,821	2	10	5	13	27	57
C24BPW37	170,213	2	6	4	7	15	34
A4BPW42	186,784	3	10	5	12	17	47
B4BPW42	175,648	3	11	5	13	31	63
C4BPW42	174,126	3	10	5	12	17	47
A8BPW42	215,745	3	8	5	8	19	43
B8BPW42	150,124	2	6	4	8	24	44
C8BPW42	150,185	3	7	4	8	18	40
A16BPW42	80,004	3	9	5	12	28	57
B16BPW42	188,075	3	7	5	8	21	44
C16BPW42	131,525	2	6	4	6	12	30
A24BPW42	152,994	3	5	5	6	15	34
B24BPW42	207,685	1	5	3	4	16	29
C24BPW42	144,153	3	7	4	9	17	40
A4EE37	166,811	3	10	5	12	21	51
B4EE37	144,627	3	10	5	15	30	63
C4EE37	148,339	3	7	4	8	17	39
A8EE37	173,640	3	6	5	6	25	45
B8EE37	143,250	3	10	5	12	30	60
C8EE37	86,123	1	3	2	3	12	21
A16 EE37	155,347	1	1	2	1	8	13
B16EE37	227,564	3	4	5	4	13	29
C16EE37	146,926	1	1	1	1	12	16
A24EE37	222,695	2	5	4	5	22	38
B24EE27	147,832	1	4	3	3	22	33
C24EE37	131,355	1	2	2	2	12	19
A4EE42	152,604	3	10	5	12	24	54
B4EE42	207,320	3	11	5	14	31	64
C4EE42	167,962	3	7	4	8	20	42
A8EE42	211,871	1	7	5	7	19	39
B8EE42	161,131	3	3	3	3	15	27
C8EE42	155,019	1	2	2	2	14	21
A16EE42	140,150	3	5	5	5	23	41
B16EE42	226,872	3	4	5	4	13	29
C16EE42	136,604	1	4	2	3	12	22
A24EE42	219,946	3	5	5	5	17	35
B24EE42	106,349	2	4	4	5	19	34
C24EE42	210,529	3	8	5	8	23	47

### Impact of BPW and EE-broth enrichment protocols on species richness, diversity, and composition of the mungo bean sprout microbial community

**Fig. 2:**
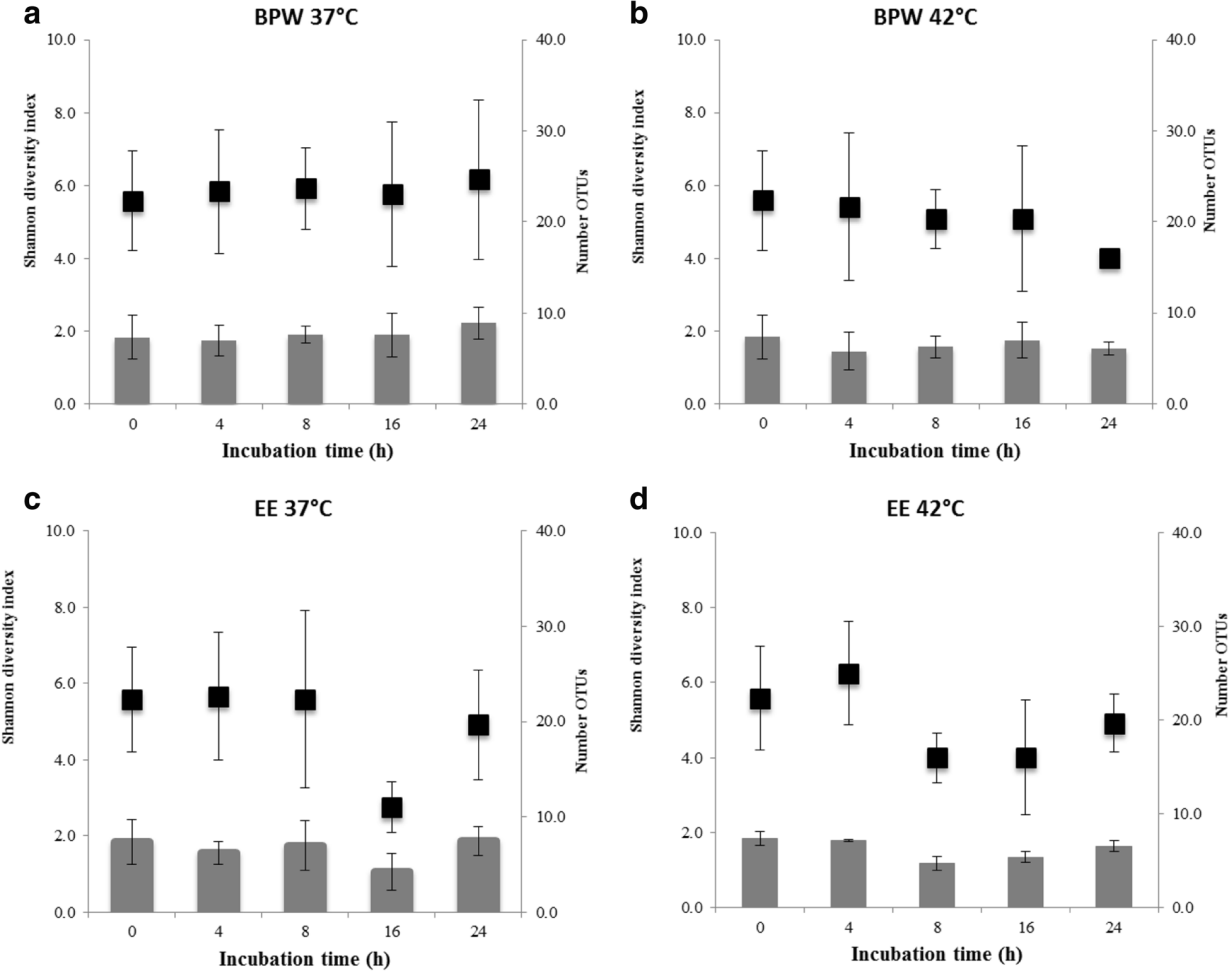
The impact of BPW and EE broth enrichment protocols on mungo bean sprout microbiome diversity at genus level based on the mean number of OTUs (97 % similarity; *black squares*) detected and Shannon diversity indexes (*grey bars*) determined in samples incubated at 37 °C (**a** and **c**) and 42 °C (**b** and **d**)

**Fig. 3:**
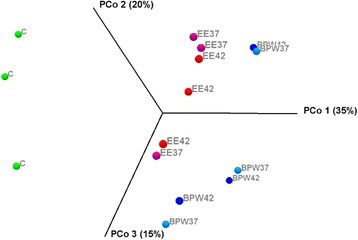
Principal coordinate analysis (PCoA) depicting differences in taxonomic composition of bacterial communities among the independent replicates of non-enriched and BPW and EE enriched (16 h) mungo bean sprout samples. Community composition dissimilarity is based on the Bray-Curtis dissimilarity metric. Percentage variation explained by each component is indicated on the axis. *Symbols* represent individual sample from the three independent biological replicates: non-enriched samples (*C*); EE broth-enriched samples incubated at 37 °C (EE 37) and 42 °C (EE 42); and BPW-enriched samples incubated at 37 °C (BPW 37) and 42 °C (BPW 42)

Species richness analysis based on the mean number of genus OTUs revealed that overall species richness in original mungo bean sprout samples did not significantly change overtime during BPW and EE-broth enrichment protocols conducted at both 37 and 42 °C (Fig. 2a–d). Although there were some notable reduction in mean species richness observed after 16-h EE-broth enrichment at both temperatures, these changes were not statistically significant (*P* > 0.05). This was partly due to the large variability observed among the independent biological replicates of same batch derived samples that were subjected to these culturing enrichment protocols. Using the Shannon diversity indices, which measure species diversity by considering both abundance and evenness, also showed that there were no significant differences in microbial community diversity between original uncultured mungo bean sprout samples and those cultured using both enrichment media and incubation temperatures at genus level (Fig. 2a–d). Meanwhile, the analysis of beta diversity revealed that there were difference in the phylogenetic composition of microbial communities between mungo bean sprout samples prior to and after enrichment for 16 h in BPW and EE-broth at both incubation temperatures (Fig. 3). Principal coordinate analysis (PcoA) based on Bray-Curtis dissimilarity analysis showed that although to begin with non-enriched samples show some level of inherent beta diversity among themselves, they still clustered together on one side of the axis independent from the 16-h culture-enriched samples. Despite some clustering of samples based on the enrichment media used, there was no clear clustering of the samples observed based on the incubation temperature conditions applied. Overall, the alpha (number of OTUs and Shannon diversity indexes) and beta diversity (PcoA) data therefore suggest that enrichment of mungo bean sprout samples in BPW and EE-broth, although not significantly changing species richness and diversity of the original sprout background microflora results in microbial communities that differ in taxonomic composition from that on uncultured mungo bean sprouts. Such changes in phylogenetic composition, however, appear to be independent of the enrichment incubation temperature (37 or 42 °C) applied.

### Identification of the mungo bean sprout bacterial community composition

**Fig. 4:**
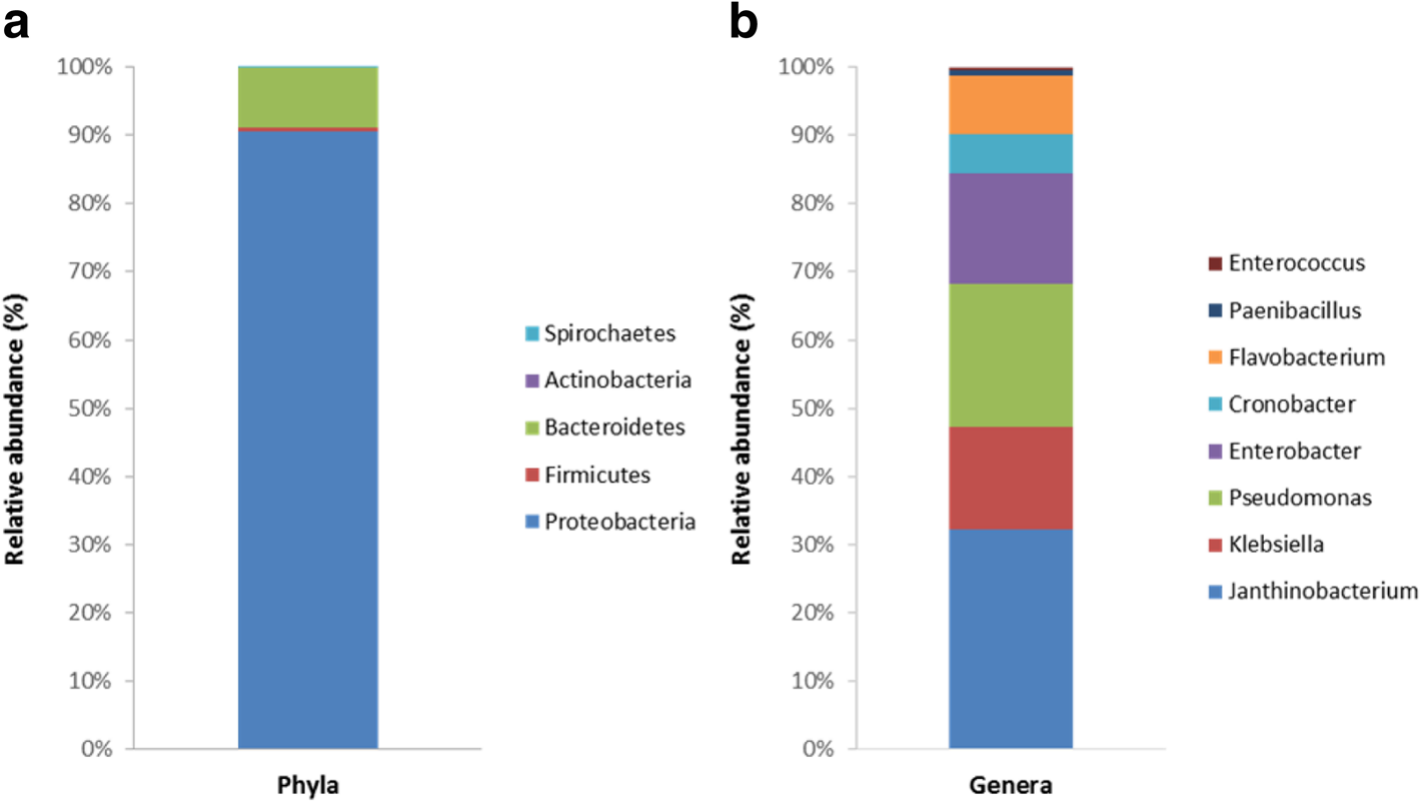
Bar charts depicting the distribution of **a** bacterial phyla and **b** dominant genera (>0.1 %) in non-enriched mungo bean sprout samples

16S rRNA sequences amplified from the uncultured mungo bean sprout samples were assigned into 5 phyla and 34 bacterial genera. An overview of the dominant phyla (>0.5 %) and genera (>0.1 %) is provided in Table 2 and Fig. 4. Based on relative abundances, the most common phyla were *Proteobacteria* (90.4 %), *Bacteroidetes* (8.8 %), and *Firmicutes* (0.6 %)**.** The most prominent genera among *Proteobacteria* sequences were *Janithobacterium* (22 %), *Pseudomonas* (14.4 %), *Enterobacter* (11.1 %), and *Klebsiella* (10.3 %)*.* While *Flavobacterium* (6.0 %) was the predominant genus from *Bacteroidetes. Paenibacillus* (0.5 %) and *Enterococcus* (0.3 %) genera represented the most predominating *Firmicutes* sequences.

**Table 2:** Mean relative abundance of dominant bacterial phyla (>0.5 %) and genera (>0.1 %) among 16S rRNA gene sequences on non-enriched mungo bean sprout controls (*t* = 0) and at various time points (*t* = 4, 8, 16, and 24 h) in BPW and EE-broth

Phylum and genus	*t* = 0	*t* = 4 h				*t* = 8 h				*t* = 16 h				*t* = 24 h			
		BPW		EE-broth		BPW		EE-broth		BPW		EE-broth		BPW		EE-broth	
		37 °C	42 °C	37 °C	42 °C	37 °C	42 °C	37 °C	42 °C	37 °C	42 °C	37 °C	42 °C	37 °C	42 °C	37 °C	42 °C
*Proteobacteria*	90.4	89.1	87.8	95.7	94.0	90.4	95.7	98.0	99.5	55.9	71.9	99.4	99.5	73.1	80.2	99.8	97.7
*Janithobacterium*	22.0	18.9	22.6	16.7	16.0	14.8	24.3	7.7	5.6	7.1	7.3	5.7	5.0	3.0	1.2	2.2	7.3
*Klebsiella*	10.3	1.1	0.5	0.6	1.7	1.3	1.7	3.2	11.9	3.9	20.7	28.5	24.5	3.8	26.6	24.6	32.3
*Pseudomonas*	14.4	7.3	4.7	9.4	9.8	3.6	4.7	9.4	2.5	1.7	2.7	1.3	1.5	2.8	0.5	1.3	1.2
*Enterobacter*	11.1	2.6	27.9	2.5	3.9	1.8	13.1	4.5	11.8	8.4	10.2	22.9	21.1	2.6	8.8	17.0	15.8
*Cronobacter*	3.8	20.7	2.8	23.2	18.5	23.1	0.0	28.8	50.3	8.1	0.01	27.6	22.1	4.3	15.1	20.1	11.9
*Duganella*	4.7	3.0	6.4	0.5	0.7	0.7	0.0	0.2	0.1	0.1	0.1	0.2	0.2	3.7	0.1	0.1	1.1
*Kosakonia*	5.1	13.7	14.9	14.5	21.9	18.4	3.6	15.7	1.2	3.5	0.5	0.4	2.8	2.2	1.0	2.6	2.1
*Leclercia*	6.8	5.6	5.6	11.5	7.1	3.6	17.8	3.3	1.5	1.3	0.9	6.1	7.0	1.1	1.3	5.1	4.9
*Acinetobacter*	3.3	3.3	1.3	0.7	0.9	2.8	0.6	0.8	0.1	7.7	7.9	0.1	0.1	20.3	16.7	0.0	0.1
*Bacteroidetes*	8.8	9.9	11.6	3.5	5.1	5.0	2.6	1.3	0.4	1.5	1.9	0.5	0.4	1.2	0.2	0.2	1.7
*Flavobacterium*	6.0	9.8	5.8	5.7	9.2	7.2	5.8	7.2	2.7	2.0	1.9	1.5	1.0	2.4	1.5	1.0	0.9
*Firmicutes*	0.6	0.9	0.6	0.8	0.9	4.6	1.7	0.7	0.2	42.6	26.2	0.07	0.05	25.7	19.6	0.02	0.6
*Bacillus*	0.0	0.0	0.0	0.0	0.0	0.2	0.0	0.9	1.4	18.9	22.8	2.4	0.0	12.8	16.4	4.8	0.0
*Paenibacillus*	0.5	2.4	0.9	2.4	1.8	4.7	0.0	4.4	0.3	3.1	0.2	0.0	0.0	0.7	0.0	0.0	5.6
*Enterococcus*	0.3	1.9	0.4	3.0	1.5	4.2	0.0	2.3	6.8	3.2	1.9	1.3	10.0	4.2	5.7	4.8	11.5

### Impact of BPW and EE-broth enrichment protocols on mungo bean sprout microbial community composition

**Fig. 5:**
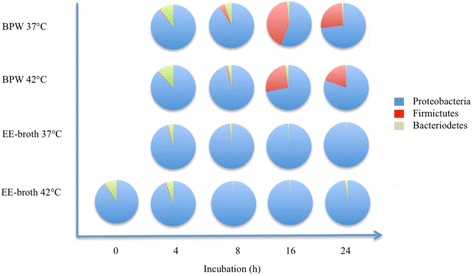
Bacterial phyla distribution of 16S rRNA sequences detected in non-cultured mungo bean sprout samples (*t* = 0) and samples collected over time (*t* = 4, 8, 16, and 24) during cultural enrichment in BPW and EE broths at 37 and 42 °C. Mean relative abundances based on three independent biological replicates of the three most abundant (>0.5 %) phyla are shown

The change in composition and abundance of the dominating phyla and genera during mungo bean sprout sample enrichment in BPW and EE-broth at 37 and 42 °C were examined. Compared to the non-cultured samples, the mean relative abundance of *Proteobacteria* increased, whilst that of *Bacteroidetes* and *Firmicutes* decreased during 24 h of culture in EE-broth samples incubated at both 37 and 42 °C (Table 2 and Fig. 5). BPW-enriched samples cultured under similar conditions on the other hand displayed a decrease in *Proteobacteria* and *Bacteroidetes* and an increase *Firmicutes* relative abundances. Such shifts in relative abundance of phyla were detectable as early as after 4 to 8 h of culture in both EE-broth and BPW-cultured samples incubated at both temperatures. Greatest shifts in relative abundance were, however, detected at 16 to 24 h of enrichment in the two enrichment media. Using the 16-h EE-broth-cultured samples as an example, the mean relative abundance of *Proteobacteria* rose from about 90 % in the non-enriched mungo bean sprout samples to above 99 % at both incubation temperatures. The abundance of this phyla decreased to less than 60 and 72 % in BPW samples cultured at 37 and 42 °C, respectively. *Bacteroidetes* abundance declined from 8.8 % in non-enriched samples to less than 2 % during 16 h of enrichment using both media independent of incubation temperature. Relative abundance of *Firmicutes* increased reaching highest levels at 16 h of enrichment at 37 °C (42.6 %) and 42 °C (26.2 %) in BPW-cultured samples. While in EE-broth-cultured samples, *Firmicutes* relative abundance declined by about tenfold (0.05–0.07 vs 0.63 %) after 16 h of culture compared to original levels detected in non-enriched microbiome of mungo bean sprout samples.

**Fig. 6:**
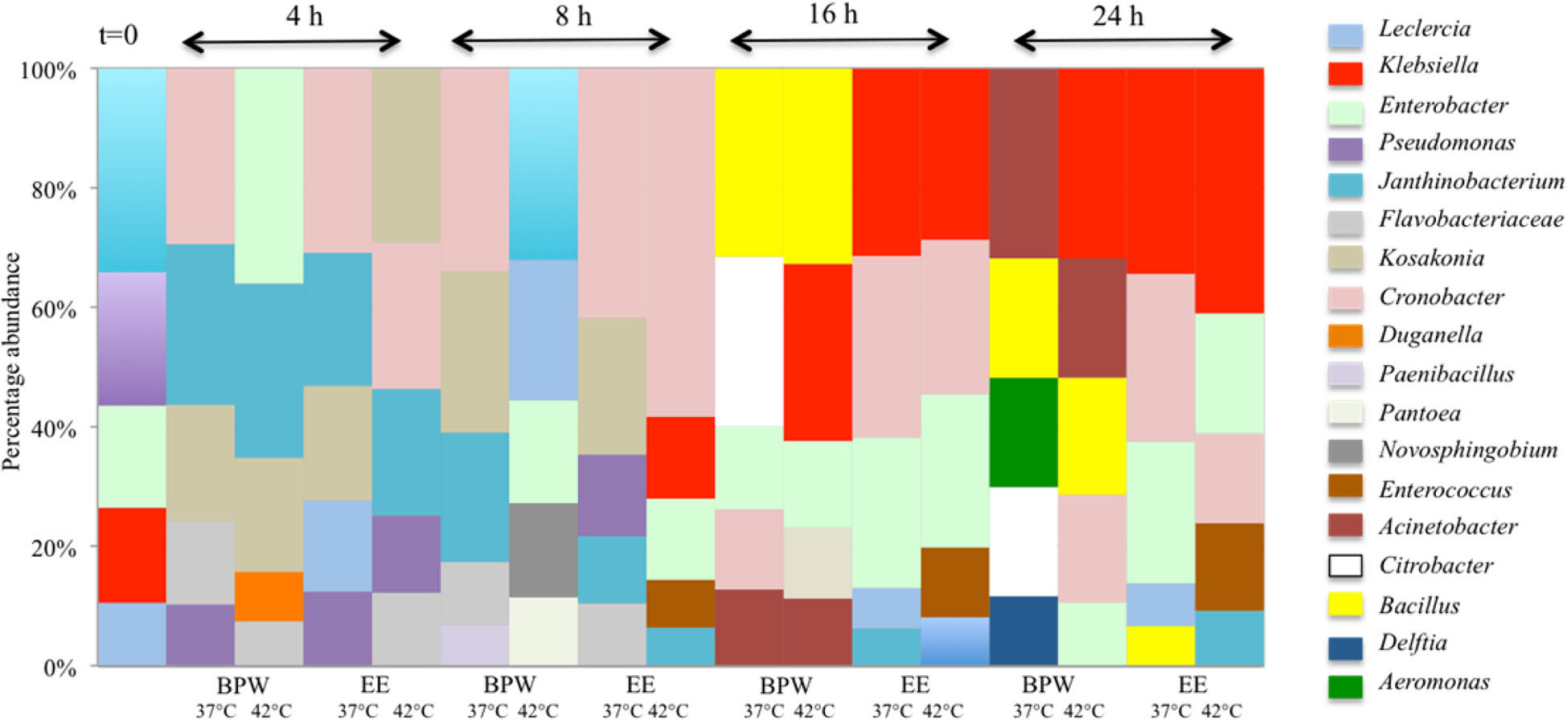
Genus level relative abundance dynamic changes over time in mungo bean sprout samples subjected to BPW and EE broth enrichment protocols during incubation at 37 and 42 °C. *Columns* represent sample means from three independent biological replicates in uncultured (*t* = 0) and enriched samples collected at various time points. *Rows* represent genus relative abundance and each cell is coloured by genera. Mean relative abundances of five most abundant bacterial genera are shown based on three independent biological experiments

*Janithobacterium* (22 %) and *Pseudomonas* (14.4 %) although the most common genera in non-enriched mungo bean sprout samples were substantially decreased by both enrichment protocols (Table 2 and Fig. 6). In samples cultured for 16 and 24 h, the *Janthinobacterium* (22 vs 1.2–7.3 %) and *Pseudomonas* (14.4 % vs 0.5–2.8 %) relative abundances were 3- to 18-fold and 5- to 28-fold decreased compared to non-enriched mungo bean sprouts, respectively. *Flavobacterium* abundance similarly decreases during enrichment in both media and at both temperatures. Meanwhile, *Klebsiella* (24.6–32-3 %) becomes the most dominant genus in all samples after 16 and 24 h of enrichment in most of the samples. The only exception being BPW enriched samples that were incubated at 37 °C, which were predominated by *Acinetobacter* (20.3 %) sequences. *Enterobacter* sequences on the other hand increased twofold (11.1 vs 21.1–22.9 %) after 16 h of culture in EE-broth. In contrast, the abundance of this genus is only slightly decreased in samples that were similarly cultured in BPW. *Bacillus* whose abundance in original mungo bean sprout samples is below the detection limit significantly increases in BPW cultured sample at both 37 and 42 °C (18.9 and 22.8 %). On contrary, its abundance in EE-broth is low (2.4 % at 37 °C), most probably due to selectivity of the medium. The level of *Enterococcus* sequences although low (0.3 %) in non-enriched sample are increased substantially (0.3 vs 10 %) in EE-broth samples cultured for 16 h at 42 °C. Notable increases (0.3 vs 4.2–5.7 %) in the abundance level of this genus were also detected after 24 h of enrichment in EE-broth at 37 °C as well as BPW at both incubation temperatures. *Paenibacillus* increases from 0.5 % prior to enrichment to 4.4–4.7 % after 8 h of enrichment in both media at 37 °C. At 42 °C in both enrichment media, *Paenibacillus* sequences almost disappear reaching only 0.3 % in EE-broth. After 16 h, the abundance of *Paenibacillus* increases to 3.1 % in BPW at 37 °C but stays very low in the other samples. After 24 h, the abundance of *Paenibacillus* is 5.6 % in EE-broth at 42 °C.

Based on Metastats analysis, statistically significant shifts (*P* < 0.05) were detected in the relative abundance of *Firmicutes*, *Acinetobacter*, *Duganella*, and *Pseudomonas* sequences between uncultured mungo bean sprout samples and those enriched 16 h in BPW (Table 3). Among samples enriched in EE-broth, significant shifts were similarly observed for *Bacteroidetes*, *Duganella*, *Kosakonia*, and *Pseudomonas* compared to the uncultured samples. Between BPW and EE-broth enriched samples, there were statistically significant differences detected in the abundance of *Firmicutes*, *Proteobacteria*, *Acinetobacter*, and *Kosakonia* sequences.

**Table 3:** Proportions of differentially abundant phyla and genera in selected groups using Metastats analysis (*P* ≤ 0.05). Samples before enrichment (*t* = 0) and samples after enrichment in either BPW or EE-broth for 16 h were compared

Genera	Phyla	*t* = 0	BPW 37 °C (16 h)	*P* value
	*Firmicutes*	0.057 ± 0.0001	0.092 ± 0.0003	0.008
*Acinetobacter*		0.032 ± 0.001	0.008 ± 0.00007	0.043
*Duganella*		0.049 ± 0.0004	0	0.005
*Pseudomonas*		0.151 ± 0.004	0.015 ± 0.0006	0.024
		*t* = 0	BPW 42 °C (16 h)	
	*Firmicutes*	0.002 ± 0.000008	0.207 ± 0.023	0.337
*Duganella*		0.024 ± 0.0001	0	0.005
*Pseudomonas*		0.074 ± 0.0009	0.009 ± 0.00006	0.014
		*t* = 0	EE 37 °C (16 h)	
	*Bacteroidetes*	0.046 ± 0.001	0.002 ± 0.000008	0.002
*Duganella*		0.024 ± 0.001	0.002 ± 0.000008	0.041
*Kosakonia*		0.027 ± 0.005	0.002 ± 0.000008	0.046
*Pseudomonas*		0.074 ± 0.0009	0.007 ± 0.0001	0.024
		*t* = 0	EE 42 °C (16 h)	
	*Bacteroidetes*	0.046 ± 0.001	0.002 ± 0.000008	0.045
*Duganella*		0.024 ± 0.0001	0	0.018
*Pseudomonas*		0.074 ± 0.0009	0.007 ± 0.00005	0.023
		BPW 37 °C (16 h)	EE 37 °C (16 h)	
	*Firmicutes*	0.231 ± 0.018	0.0003 ± 0.0001	0.021
	*Proteobacteria*	0.286 ± 0.017	0.499 ± 0.0000005	0.015
*Acinetobacter*		0.039 ± 0.0000009	0.0002 ± 0.00000004	0
*Kosakonia*		0.018 ± 0.00005	0.002 ± 0.000005	0.01
		BPW 37 °C (16 h)	BPW 42 °C (16 h)	
*Kosakonia*		0.018 ± 0.00008	0.002 ± 0.0000005	0.028

**Fig. 7:**
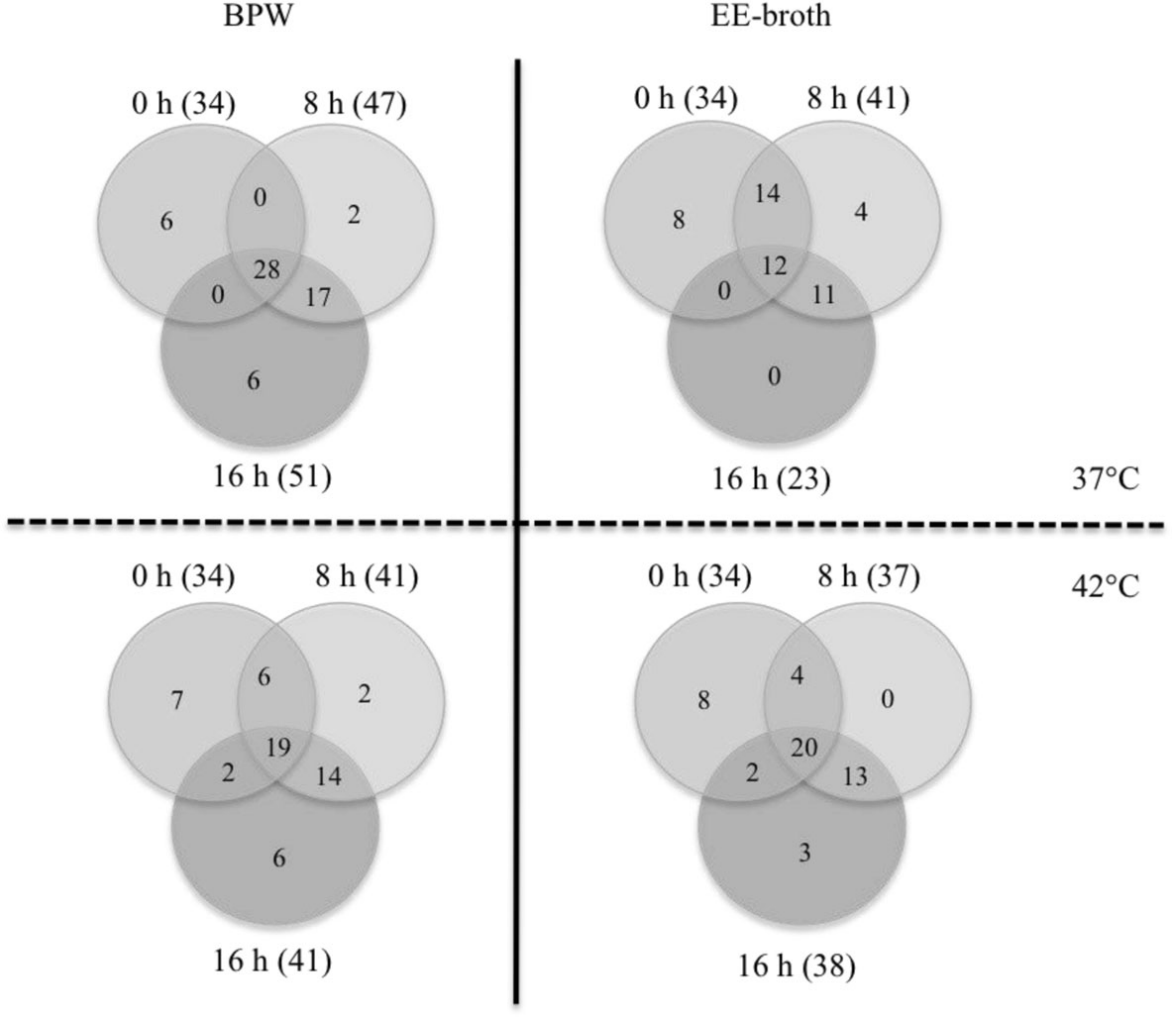
Venn diagrams showing the distributions of unique and shared genera between mungo bean sprout microbial communities before (0 h) and after 8- and 16-h enrichment in BPW at 37 and 42 °C and EE-broth at 37 and 42 °C

Since current BPW and EE-broth enrichment protocols generally use incubation periods ranging from 16 to 18 h, the OTU composition at genus level of the non-enriched mungo bean sprout samples were also compared to those enriched for 8 and 16 h in these two media at 37 and 42 °C (Fig. 7). The sprout microbiome composition changes the least during BPW enrichment at 37 °C for 16 h. Under these conditions, the greatest number (28/59; 47 % of all genera in these samples) of shared genera with the uncultured samples was detected. In contrast, similarly incubated EE-broth enriched samples displayed the least the number of genera (12/49; 24.5 % of all genus OTUs) that were common with the uncultured samples and the rest of samples enriched for 16 h. This indicates that the composition of 16-h EE-broth enriched samples differed the most compared to the other samples. The numbers of genera shared between uncultured mungo bean sprout samples and both BPW (19) and EE-broth (20) enriched samples for 16 h at 42 °C were comparable. When non-enriched samples are compared to samples enriched for 8 h, the highest and lowest numbers of shared genera were observed in EE and BPW broths at 37 °C, respectively. This suggests that in BPW, the greatest changes in bacterial composition take place in the beginning of the enrichment whereas in EE-broth, the shifts in composition occur after 8 h.

## Discussion

Despite various efforts that have previously been undertaken to improve the current methods for detection of foodborne pathogens from sprouted seeds, there are various difficulties that still remain [19–22]. One drawback in particular is the lack of physiological traits that would allow the selective discrimination of target pathogens such as STEC and *Salmonella* from large amounts of sprout associated background flora predominated by non-target bacteria including enterobacteria species that outnumber these pathogens by several orders of magnitude. In addition, we currently lack detailed knowledge concerning the genetic composition of sprout associated microbial flora since previous investigations largely focused on determining quantitative aspects of the enrichment process on target pathogens in sprouts. BPW is an unselective medium applied for the enrichment of pathogens from food samples. EE-broth is a selective medium that enriches for Enterobacteriaceae while inhibiting growth of non-Enterobacteriaceae microorganisms. We used next-generation sequencing to determine the baseline composition of microbial species comprising the mungo bean sprout microbiome and to assess how it changes during BPW- and EE-broth-based enrichment protocols used for detection of Gram-negative foodborne pathogens.

Our initial culture based enumeration of the mungo bean sprout background flora on non-enriched samples showed relatively high APC (6.9 + 0.81 log cfu/g) values in agreement with observations made by others on different sprouts varieties and leafy greens [23, 24]. The culturable background flora included *Klebsiella*, *Citrobacter* and *Pantoea* species**.** We observed that the APC counts of this background flora displayed similar growth kinetics over 24 h during BPW and EE-broth enrichment protocols. More specifically independent of these two enrichment media and incubation at 37 °C or 42 °C, the APC values of the mungo bean background flora increased from about 7 to 10 log cfu/ml after a 24-h enrichment.

In order to gain more insights into the genetic composition of the mungo bean sprout-associated microbial flora, we applied 16S rRNA-based sequencing. We first determined the baseline microbiome composition on mungo bean sprouts and then assessed how it changed during enrichment protocols based on BPW and EE-broth. The mungo bean sprout baseline microbiome was dominated by *Proteobacteria* (90.4 %) but also included *Bacteroidetes* (8.8 %) and *Firmicutes* (0.6 %) sequences*.* Our findings are similar to those of previous studies in alfalfa sprouts demonstrating that the microbiome was predominated by sequences of the phylum *Proteobacteria* [21]. The examination of the mungo sprout microbiome data at genus level showed the predominance of *Janthinobacterium*, *Klebsiella*, *Pseudomonas*, *Enterobacter*, and *Cronobacter* among the *Proteobacteria* sequences. *Flavobacterium* was the most common *Bacteroidetes* genus, whilst *Firmicutes* sequences were predominantly associated with *Paenibacillus* and *Enterococcus* genera. Interestingly, the *Flavobacterium* genus includes various psychrophilic species probably reflecting the impact of sprout distribution and storage at low temperatures in shaping the sprout microbiome composition. The overall number of OTUs detected in the mungo bean samples was comparable to values reported in other sprouted seeds [25] but lower compared to those reported in other produce such as rocket salad or oak leaf lettuce [26, 27]. The lower number of genera on sprouted seeds can be explained by their soil free production as well as by the fact that the process of drying the seeds probably reduces the associated flora to more resistant representatives.

No quantitative differences in the aerobic mesophilic colony counts were discerned between the different enrichment media and incubation temperatures applied. In contrast, clear differences in microbiome composition associated with the BPW and EE-broth enrichment protocols were revealed through microbiome sequencing. BPW enrichment is the prescribed ISO protocol for the detection of STEC in sprouts. We observed here that enrichment of mungo bean sprout samples using this non-selective media actually raises the relative abundance of *Firmicutes* while decreasing *Proteobacteria* and *Bacteroidetes* abundances compared to relative abundances detected on non-enriched sprout samples. Such trends in microbiome composition shift were largely preserved in enrichments performed at 37 and 42 °C. In addition, such phylum relative abundance shifts in the microbiome changes were accompanied by various genera specific composition changes. For example, BPW enrichment microbiome observed at 16 h revealed that among the *Firmicutes*, the predominant genera shifted from *Paenibacillus* (0.5 %) and *Enterococcus* (0.3 %) to *Bacillus* (18.9 %). Although this genus is below the detection level in the uncultured mungo bean sprout microbiome. BPW enrichment also shifted the *Proteobacteria* towards *Enterobacter* (37 °C) and *Klebsiella* (42 °C) predominance depending on the incubation temperature. Relative abundance of these two genera increases to surpass those of *Janithobacterium* and *Pseudomonas* that represent *Proteobacteria* sequences with the highest relative abundances in the uncultured mungo bean sprout microbiome.

EE-broth enriches for enterobacteria while inhibiting non-enterobacteria species including Gram-positive bacteria, a desirable outcome when aiming to enrich for Gram-negative pathogens from many types of food products. As expected, we found that enrichment with this broth pushes the microbiome towards enterobacteria. This results in an almost complete predominance of *Proteobacteria* that account for more than 97.7 % of the microbiome sequences compared to 94.4 % in the non-enriched mungo bean sprout microbiome. Relative abundances of *Firmicutes* and *Bacteroidetes* sequences on the other hand are reduced below levels that were detected prior to EE-broth culture of the mungo bean sprouts. These trends were not strongly influenced by the incubation temperature. More significantly, however, was the observation that EE-broth enrichment induced shifts in the relative proportions of various *Proteobacteria* genera compared to the non-enriched mungo bean sprout microbiome. Notable changes were that *Janithobacterium* and *Pseudomonas*, genera that predominated in the non-enriched mungo spout microbiome decrease in relative abundance, whereas *Klebsiella*, *Cronobacter*, and *Enterobacter* increase in relative abundance to become the predominant genera in the 16- and 24-h EE-broth enriched samples.

Overall, we observed that the incubation temperatures (37 or 42 °C) did not contribute to substantial differences in the composition of the enriched microbiome. There were, however, a few genera whose relative abundance was strongly influenced by the incubation temperature. We observed for example that BPW enrichment performed at 42 °C enhanced the relative abundance of the genus *Klebsiella* (20.7 vs 3.9 %) compared to 37 °C. While BPW enrichment performed at 37 °C enhanced on the other hand *Cronobacter* (8.1 vs 0.1 %) and *Paenibacillus* (3.1 vs 0.2 %) abundances compared to 42 °C. The phylum as well as genera specific shifts during mungo bean sprout enrichment could be explained by selective growth, death, or survival of microorganisms of the mungo bean sprout microbiome as conditions in the enrichment media changed. Alternatively, disproportionate increase in the concentration of DNA templates representing the predominating organisms might have consequently pushed templates from less predominant organisms below the detection limit of the 16 rRNA sequencing depth applied.

Although the observation that EE-broth favours enrichment of the phylum *Proteobacteria* including pathogens such as STEC and *Salmonella* should be a desirable trait of this media, we have also previously observed that the growth of STEC spiked into mungo bean sprouts terminates prematurely thereby hindering their detection in contaminated sprouts [12]. Based on this, we concluded that this was probably due to competition and outgrowth of the STEC by non-target enterobacteria species that occur at high abundances within the mungo bean sprout background flora. Alternatively, some enterobacteria species of the mungo bean microbiome might grow more efficiently pushing the level of target pathogens below the limit of the subsequent PCR-based detection assays. Microbiome analysis showed that EE-broth enrichment, although selectively enriching for *Proteobacteria*, has clear genus-specific impacts. Genera such as *Klebsiella*, *Enterobacter*, and *Cronobacter* increased in relative abundance whereas others such as *Janithobacterium* and *Pseudomonas* decreased after EE-broth-based enrichment. It might be therefore that enrichment of pathogens such as STEC that occur at low concentrations is likewise hindered due to competition for nutrients from other more predominant *Proteobacteria* genera of the mungo bean sprout microbiome. It could also be that such pathogens are less favoured by the conditions generated in the media during the EE-broth enrichment protocol as observed for the other *Proteobacteria* genera. These aspects will, however, require further investigations including microbiome analysis that will monitor the behaviour of pathogens during enrichment with respect to other organisms of mungo bean sprout-associated microbiome.

BPW, the prescribed enrichment medium for STEC detection in sprouts, was found to shift the microbial community towards an increased proportion of *Firmicutes* while decreasing the proportion of the *Proteobacteria* phyla that also includes STEC. Similar observations were also previously described during enrichment of *Salmonella* in cilantro and tomato [13, 14]. In addition, among *Firmicutes*, the genus *Paenibacillus* was shown to inhibit and kill the *Proteobacterium Salmonella* in the enrichment of the tomato phyllosphere. In fact, a strain of *Paenibacillus* has even been proposed for use as an antimicrobial against pathogens such as *Escherichia*, *Listeria*, and *Shigella* [14, 28]. *Paenibacillus* sequences were detected in uncultured mungo bean sprout samples indicating that it also constitutes their original microflora. More importantly, *Paenibacillus* relative abundance increased and ranks among the 20 most dominant genera detected in 8- and 16-h BPW-enriched mungo bean sprout samples cultivated at 37 °C. Increased abundance of this genus might be therefore among factors contributing to repressed growth of pathogens such as *Salmonella* and *E. coli*, if they are present on the mungo bean sprout phyllosphere during BPW based enrichment. The impact of various other genera comprising the sprout microbiome on growth of pathogens such as STEC is not yet known.

Meanwhile, another possible cause of the cessation of STEC growth observed during enrichment might be nutrient depletion. Growth suppression of multispecies bacterial populations in batch cultures by a dominant group of bacteria is known as the “Jameson effect” [29]. Two bacterial populations inoculated into a medium or food matrix will grow in numerical parity similar to their initial proportion until stationary phase of the numerical dominant population is reached, which is proposed to be determined by the limitation of nutrients or the accumulation of toxic compounds [30]. Inhibiting growth of the most dominant representative should therefore limit the deprivation of nutrients and facilitate growth of Gram-negative foodborne pathogens.

## Conclusions

New insights into the mungo bean sprout microbiome and its changes during the BPW and EE-broth enrichment protocols used for detecting Gram-negative pathogens were generated. Based on aerobic mesophilic colony counts, the mungo bean sprout background flora did not show significant differences in growth kinetics during the two enrichment protocols and at different incubation temperatures. Microbiome sequencing, however, revealed that there were huge changes induced to the composition of the mungo bean sprout microbiome following BPW- and EE-broth-based enrichment protocols that were largely not dependent on the incubation temperatures. The uncultured mungo bean sprout microbiome is largely dominated by sequences from the phylum *Proteobacteria* followed by those from *Bacteroidetes* and *Firmicutes*. BPW enrichment reduced the relative abundance of *Proteobacteria* while increasing the level of *Firmicutes* compared to the original mungo bean sprout microbiome. EE-broth enrichment on the other hand preserved and increased the relative abundance of *Proteobacteria* sequences while decreasing the abundance of *Firmicutes*. Both enrichment protocols are accompanied by various genera-specific changes. Overall, the changes induced in the mungo bean sprout microbiome during these two enrichment protocols might have some negative consequences for the detection of Gram-negative pathogens that usually occur at low concentrations in sprouts.

## Abbreviations

APC: Aerobic plate counts
BPW: Buffered peptone water
EE-broth: Enterobacteriaceae enrichment broth
OTU: Operational taxonomic unit
PcoA: Principal coordinate analysis
STEC: Shigatoxin-producing *E. coli*
